# Development of novel lipoplex formulation methodologies to improve large-scale transient transfection for lentiviral vector manufacture

**DOI:** 10.1016/j.omtm.2024.101260

**Published:** 2024-04-26

**Authors:** Thomas Williams-Fegredo, Lee Davies, Carol Knevelman, Kyriacos Mitrophanous, James Miskin, Qasim A. Rafiq

**Affiliations:** 1 Oxford Biomedica (UK) Limited, Windrush Court, Transport Way, Oxford OX4 6LT, UK; 2 Advanced Centre for Biochemical Engineering, Department of Biochemical Engineering, University College London, Bernard Katz Building, Gower Street, London WC1E 6BT, UK

**Keywords:** lentiviral vector, transient transfection, transient gene expression, HEK293T, cationic liposomes, lipoplexes, process development, cell and gene therapy, scale-up

## Abstract

Large-scale transient transfection has advanced significantly over the last 20 years, enabling the effective production of a diverse range of biopharmaceutical products, including viral vectors. However, a number of challenges specifically related to transfection reagent stability and transfection complex preparation times remain. New developments and improved transfection technologies are required to ensure that transient gene expression-based bioprocesses can meet the growing demand for viral vectors. In this paper, we demonstrate that the growth of cationic lipid-based liposomes, an essential step in many cationic lipid-based transfection processes, can be controlled through adoption of low pH (pH 6.40 to pH 6.75) and in low salt concentration (0.2× PBS) formulations, facilitating improved control over the nanoparticle growth kinetics and enhancing particle stability. Such complexes retain the ability to facilitate efficient transfection for prolonged periods compared with standard preparation methodologies. These findings have significant industrial applications for the large-scale manufacture of lentiviral vectors for two principal reasons. First, the alternative preparation strategy enables longer liposome incubation times to be used, facilitating effective control in a good manufacturing practices setting. Second, the improvement in particle stability facilitates the setting of wider process operating ranges, which will significantly improve process robustness and maximise batch-to-batch control and product consistency.

## Graphical abstract

**Figure:**
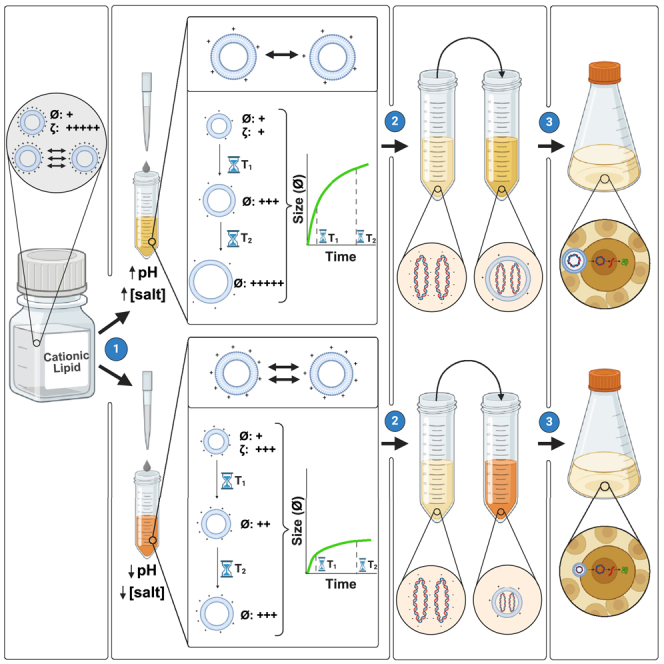

## Teaser

Rafiq and colleagues have developed several improved transfection methodologies for the production of LVVs, leading to improvements in process robustness and process control in a manufacturing setting. These solutions address existing challenges related to large-scale transient transfection which presently complicate the GMP manufacture of viral vectors.

## Introduction

Cell and gene therapies (CGTs) represent transformative treatments that are able to modify and alleviate the underlying genetic causes of diseases in a variety of approaches, potentially providing curative treatments for patients rather than ameliorating the symptoms of many debilitating and intractable diseases.^1^ These therapies have the potential to target complex diseases that presently have no effective treatments, representing a new frontier in medicinal innovation. The development of technologies enabling efficient transient gene expression (TGE) has been crucial to the progress of the CGT field, evolving TGE into an attractive industrial technology capable of facilitating rapid production of a diverse range of biologics, including viral vectors, in the quantities required for clinical development and commercial manufacture.^2^ The utilization of suspension-adapted cells, such as human embryonic kidney (HEK)293 lines, and the availability of highly efficient synthetic transfection reagents have enabled large-scale transient transfection to become a successful and effective strategy to produce biopharmaceutical products.^3^

TGE technologies, traditionally associated with the generation of preclinical and research-grade material, have significantly advanced in the last 20 years, and there are now many examples of transient transfection processes which have been successfully scaled-up to the hundred and thousand liter scale.^4^^,^^5^^,^^6^^,^^7^ Most gene therapy viral vectors used commercially and in clinical trials are manufactured via TGE.^8^ However, insufficient manufacturing capacity, due to the requirement of complex specialist facilities and complex manufacturing processes required for viral vector production, has resulted in a global shortage of viral vectors.^9^ By 2025, the U.S. Food and Drug Administration anticipates the approval of between 10 and 20 new CGT products per year,^10^ meaning that viral vector demand will continue to exceed the present global production capabilities as more therapies reach late-stage clinical trials and are commercialized. To ensure that demand is addressed, it is crucial that improved transfection technologies and processes are developed to ensure that TGE-based bioprocesses can consistently manufacture large quantities of commercial-grade viral vectors within appropriate tolerances for infectivity/potency, purity, and safety, while ensuring high levels of regulatory compliance.

Chemical transfection methodologies represent the most widely used technique for facilitating exogenous gene transfer.^11^ The fundamental principle behind synthetic nucleic acid delivery vectors is similar; these reagents coat nucleic acid (DNA or RNA), condensing it into nanoparticles that commonly exhibit a net neutral or net positive charge, thereby allowing these complexes to come into close proximity with negatively charged cell membranes to facilitate efficient gene delivery.^12^ Synthetic cationic lipids and cationic polymers have largely superseded traditional chemical transfection reagents, such as calcium phosphate, and are extremely popular due to their effectiveness *in vitro* and limited cytotoxicity.^11^^,^^13^ Lipid-based transfection reagents are generally reported to achieved higher transfection efficiencies in immortalized human and animal cell lines when compared with non-liposomal-based reagents, including cationic polymers. However, cationic polymers, including the commonly used polyethylenimine (PEI), are widely reported to exhibit decreased cytotoxic effects on host cells when compared with lipofection.^14^ The transfection efficiency and cytotoxicity associated with different transfection reagents can vary depending on the specific cell line and experimental conditions and optimization experiments to determine the most suitable transfection system are recommended. Cost is also an important consideration, particularly for larger scale processes, and PEI is cheaper than many lipid-based reagents;^15^ however, the transfection performance attained should be considered when determining which reagent is more cost effective for a particular bioprocess. Synthetic cationic lipids, which form the focus of this work, form colloidal structures, termed liposomes, in aqueous solutions. The preparation of liposomes constitutes a critical and sensitive intermediate process step in the generation of many cationic lipid-based transfection complexes. This includes Lipofectamine brand reagents, which are recognized as one of the most-cited transfection reagent families and are widely considered to be the gold standard for *in vitro* gene delivery.^16^^,^^17^ The subsequent electrostatic interactions between synthetic cationic liposomes and polyanionic nucleic acid result in the spontaneous formation of nucleic acid-cationic lipid complexes, termed lipoplexes, which facilitate lipid-mediated transfection (lipofection).^18^^,^^19^

One major challenge associated with large-scale TGE-based bioprocessing is the ability to achieve reliable and efficient delivery of nucleic acid into cells to ensure high levels of TGE.^20^^,^^21^ This challenge is particularly pronounced in viral vector manufacturing processes due to the necessity to simultaneously co-transfect cells with multiple plasmids.^22^ Transfection processes are multifactorial and highly sensitive to factors related to the intrinsic physiochemical and electrochemical properties of lipoplexes, as well as factors related to the specific cell culture bioprocessing conditions that are employed, all of which can impact process performance.^23^ Consequently, complications are often encountered when attempting to scale TGE processes from research and development-scale to industrial manufacturing scale when following contemporary lipoplex preparation methodologies.^24^ Among the challenges of scaling such processes, there are three key issues associated with preparing transfection mixtures for hundred liter scale bioprocesses that are related to liposome incubation time, complex stability, and transfection mixture volume that are encountered when employing standard lipoplex preparation methodologies. First, manufacturer’s recommended liposome incubation times, which correlate with efficient transfections, are often not practically attainable in the context of hundred liter scale transfection processes. Second, liposomes can exhibit poor stability when prepared according to contemporary protocols. Third, transfection mixes are typically large volumes and a large proportion (approximately 10%^6^^,^^25^^,^^26^) of the overall culture volume being transfected; such preparations are logistically challenging to prepare in a large, industrial-scale, manufacturing setting. We present three alternative liposome/lipoplex formulation methodologies that seek to address the issues identified when employing traditional transfection protocols for the large-scale manufacture of biologics via TGE.

## Results

### Liposomal growth kinetics can be controlled by regulating the formulation pH, salt concentration, and liposome concentration

#### Controlling liposomal growth kinetics by regulating formulation pH

Lipoplex particle size and surface charge are critical determinates of transfection efficiency and liposomes have been shown to rapidly aggregate when added to cell culture medium.^27^^,^^28^^,^^29^^,^^30^^,^^31^ The salt concentration in the culture medium disrupts the electrostatic repulsive forces between adjacent liposomes, destabilizing them, allowing liposomes to come into closer contact and aggregate into larger particles.^32^^,^^33^ To determine whether the hydrodynamic size and growth rates of liposome particles, prior to their complexion with plasmid DNA (pDNA), were influenced by the pH of the formulation used to prepare the nanoparticles, Lipofectamine 2000CD-based liposome particle growth was measured via dynamic light scattering (DLS) in a series of pH adjusted FreeStyle 293 Expression Medium aliquots. pH was adjusted to the target value via the addition of hydrochloric acid and NaOH, as appropriate.

**Figure 1:**
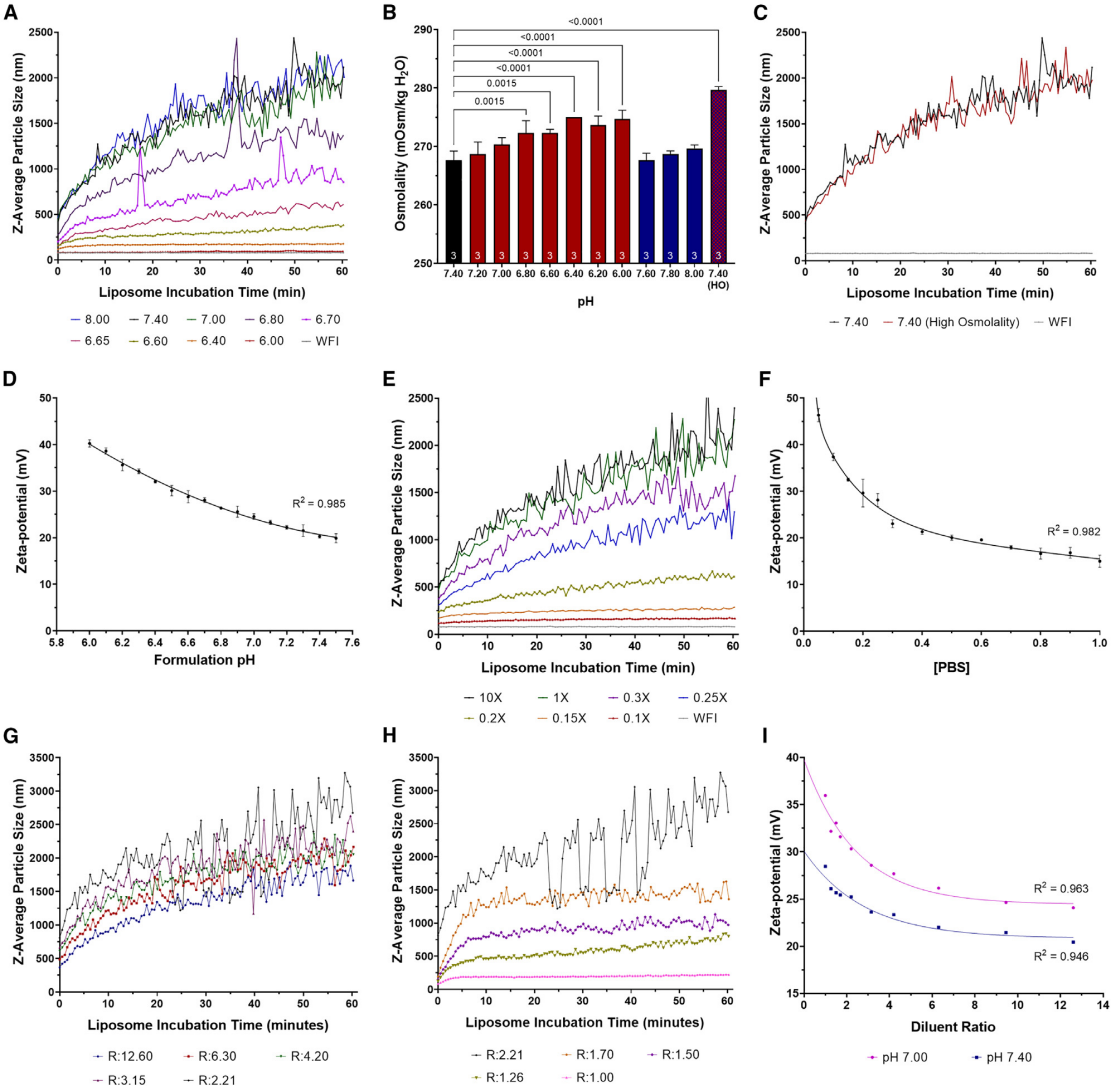
Lipofectamine 2000CD liposome particle characterization studies (A) Liposome growth profiles measured in pH-adjusted FreeStyle 293 Expression Medium between a pH range of 6.00 and 8.00. (B) Osmolality of pH adjusted FreeStyle 293 Expression Medium following 2 M hydrochloric acid (HCl) (red bars) or 0.5 M sodium hydroxide (NaOH) (blue bars) addition and a high osmolality (HO) FreeStyle variant. Data represents the mean ± one SD (n = 3, replicates indicated at bottom of bars) and statistically significant pairwise comparisons have been presented as *p* values. (C) Liposome growth profiles measured in FreeStyle medium compared with the FreeStyle HO medium variant. (D) Relationship between liposome zeta-potential and FreeStyle 293 formulation pH between a pH range of 6.00–7.50. Each data point is the average of triplicate conditions and error bars are ±1 SD of the mean. (E) Liposomal particle growth profiles in varying concentrations PBS. (F) Relationship between liposome zeta-potential and PBS concentration. Each data point is the average of triplicate conditions and error bars are ±1 SD of the mean. (G) Liposome particle growth measured in varying liposome diluent ratios initially resulting in increased particle growth rates and (H) then in reduced particle growth rates. (I) Relationship between liposome diluent ratio, formulation pH and zeta-potential. See also Figure S1.

Analysis of the DLS data showed that the liposomal growth rates were equivalent between a pH of 7.40–8.00 (Figure 1A). Under these conditions, particles reached a size of 1,000 nm after an incubation period of approximately 8 min and 1,500 nm after a 25-min period. Reducing the pH of the medium below a pH of 7.40 resulted in reduced rates of particle growth; particle growth slowed with decreasing pH. At a pH of 6.00, no liposomal growth was detected in 60 min, and the nanoparticles maintained a comparable size to those incubated in water for injection (Figure 1A).

To control for the potential impact that the increased osmolality of the pH adjusted FreeStyle medium may be having on liposome particle growth, a high osmolality FreeStyle medium variant was generated. This involved the initial pH adjustment of FreeStyle from a starting pH of 7.40 to 6.00 and the subsequent pH adjustment of the medium from a pH of 6.00 back to 7.40 (Figures 1B and 1C). An equivalent particle growth rate was measured following the addition of Lipofectamine 2000CD to unmodified FreeStyle medium (267.7 mOsm/kg H_2_O) and the high osmolality FreeStyle variant (279.7 mOsm/kg H_2_O), when both were maintained at pH 7.40. This suggested that the increased osmolality of the various test conditions was not influencing particle growth kinetics in this particular context and that pH is a principal parameter governing liposome growth rate.

Lipoplex size is a major determinant of transfection efficiency^27^^,^^34^ and lipoplexes of a diameter ranging from 400 nm to 1,400 nm have been shown to yield high transfection efficiencies compared with smaller or larger particles.^35^ It was hypothesized that incubating liposomes in low pH formulations, resulting in decreased particle growth rates, would delay the onset of peak particle performance, as particles would take longer to reach the required optimal size that correlate with efficient transfection. Peak particle performance in this context is defined as liposome particle properties that ensure high transfection efficiencies, high levels of TGE, and high functional titers when generated liposomes are utilized for the production of LV vectors.

It is well established that the zeta-potential, defined as the potential difference between the surface of a colloidal particle in a conducting liquid suspension and the bulk fluid, is a determinant of transfection efficiency.^36^ It was hypothesized that the zeta-potential of liposomes was the key driving force behind the differential particle growth rates observed across the pH range investigated. Liposome zeta-potential was found to vary significantly with formulation pH. Nanoparticles prepared at a pH of 7.50 exhibited an average zeta-potential of +19.9 mV compared with a zeta-potential of +40.3 mV when prepared at a pH of 6.00 (*p* < 0.0001) (Figure 1D). No detectable growth of particles was measured when liposomes were incubated at a pH of 6.00 indicating that, at a zeta-potential of +40.3 mV, liposomes exhibit sufficient repulsive electrostatic forces to prevent particle coagulation.

#### Controlling liposomal growth kinetics by regulating formulation ionic strength

Another factor that can significantly influence transfection efficiency is the specific salt composition and ionic strength of formulations used to prepare cationic lipid-based transfection complexes.^37^ The impact of the ionic strength on liposome hydrodynamic size and particle growth rates, prior to their complexion with pDNA, was determined. Lipofectamine 2000CD-based liposome particle growth was measured via DLS in a series of PBS solutions with differing salt concentrations. The salt concentration of the various formulations was adjusted to the target concentration via the dilution of a 10× PBS stock solution in water. The liposomal growth rate was equivalent in solutions maintained at a concentration of 10× PBS to 1× PBS. Particles reached a size of 1,000 nm after an incubation period of approximately 8 min and a size of 1,500 nm after a 25-min period, demonstrating an equivalent growth rate to liposomes incubated in FreeStyle medium (Figure 1E). Decreasing the salt concentration of PBS below 1× PBS resulted in lower rates of particle growth that further decreased with lower salt concentrations. At a salt concentration of 0.1× PBS, minimal liposomal growth was detected with particles achieving a maximum size of 167 nm after a 60-min incubation. While not actively controlled, the pH of each of the formulations was measured to ensure comparable pH readings were obtained in all solutions, necessary since pH was earlier shown to influence liposomal growth rate. Ionic strength is known to influence particle surface charge, which likely accounts for the observed lower liposomal growth rates when particles were prepared in low salt concentration formulations, since the behavior of aqueous dispersions is highly sensitive to the ionic structure of the interface between the colloidal particles and the liquid.^38^ The zeta-potential of liposomes in differing concentrations of PBS, ranging from 0.05× to 1× was assessed and was found to vary significantly with salt concentration (Figure 1F). Nanoparticles prepared in 1× PBS exhibited an average zeta-potential of +15.0 mV compared with a zeta-potential of +46.3 mV when prepared in 0.05× PBS (*p* < 0.0001).

#### Controlling liposomal growth kinetics by regulating the formulation temperature

Since zeta-potential has also been shown to be influenced by temperature,^39^ Lipofectamine 2000CD-based liposome particle growth and zeta-potential were measured in FreeStyle 293 Expression Medium maintained at 1°C, 5°C, and 23°C to determine whether growth rates could be decreased at lower temperatures. Equivalent zeta-potentials were measured for nanoparticles prepared at all temperatures evaluated and similar rates of particle growth were observed for particles prepared at 1°C and 5°C, compared with 23°C, with only a modest decrease in the growth rate being observed (Figure S1).

#### Controlling liposomal growth kinetics by regulating the liposome concentration

To assess the feasibility of preparing Lipofectamine 2000CD-based liposomes at high concentrations, nanoparticles were prepared in varying volumes of FreeStyle 293 Expression Medium. Particle growth rates were found to be strongly influenced by the volume of the diluent (Figures 1G and 1H). Initially, particle growth rates were found to increase when prepared at higher concentrations. However, above a threshold concentration factor, corresponding with a diluent:liposome ratio of 2.21:1, liposomal growth rates decreased, indicating that there is a concentration limit above which transfection performance is likely to deteriorate. Liposome zeta-potential was found to increase as the concentration of the nanoparticles was increased, likely explaining the lower growth rates that are observed above a threshold value (Figure 1I). Initially, increasing the concentration of the liposomes resulted in adjacent particles residing in a closer proximity to each other. This reduced distance likely explains the higher particle growth rates, as there is an increased probability of neighboring particles interacting more frequently. However, as the particle concentration and zeta-potential increase, a critical threshold value seems to be reached, likely resulting in increased repulsive electrostatic forces between adjacent particles and lower growth rates. This critical value will likely be determined by the precise nature of the formulation that serves as the liposome diluent, as different formulations will have varying ionic strengths, resulting in different zeta-potentials being assigned to particles under different concentrations.^40^

### Preparing lipoplexes at low pH and in low salt concentration formulations enhances complex stability and delays the onset of peak particle performance

The incubation period of cationic lipid-based transfection reagents in aqueous formulations prior to DNA complexion, as well as the post-complexion incubation period of lipoplexes prior to their addition to cells, impacts transfection efficiency.^27^ Manufacturers of certain cationic lipid-based transfection reagents advise the utilization of shorter liposome incubation periods (approximately 5 min) to avoid decreased transfection performance.^41^^,^^42^ This represents a significant challenge when preparing lipoplex-based transfection mixtures for large-scale (>100 L) bioprocesses, as short incubation times are very challenging to achieve practically and with sufficient process control and flexibility.

It was hypothesized that incubating liposomes in low pH formulations, resulting in decreased particle growth rates, would delay the onset of peak particle performance, as particles would take longer to reach the required optimal size that correlates with efficient transfections. The impact of formulation pH and liposome incubation time on transfection performance was investigated, using transfection efficiency and the median fluorescence intensity of transfected cell populations as the key output metrics. A three-level, face-centered central composite response surface (fcCCD) design of experiment (DoE) was utilized to examine the impact of the selected variables on transfection performance.

**Figure 2:**
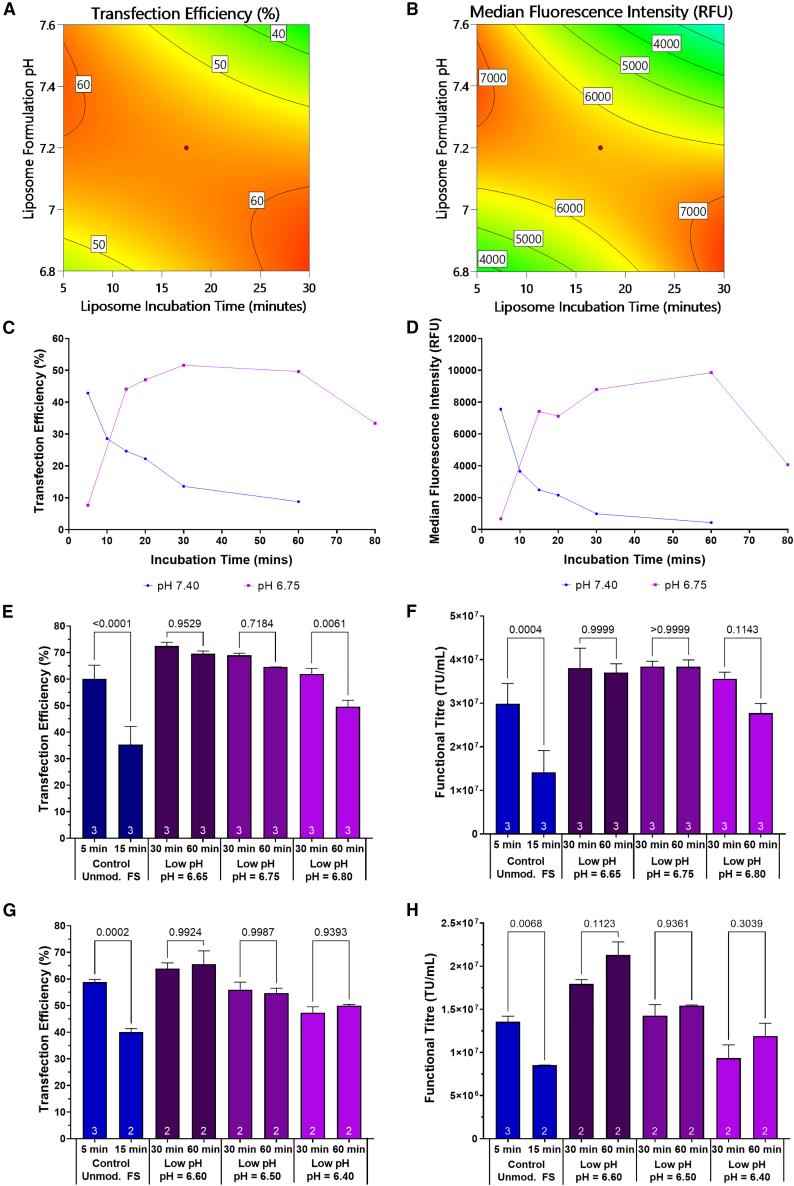
Assessment of lipoplex performance and stability when preparing nanoparticles in formulations of varying pH (A) Two-dimensional contour maps of the design space modeled for the impact of FreeStyle 293 pH and liposome incubation time on transfection efficiency and (B) MFI, measured 24 h after transfection. (C) Transfection efficiency and (D) MFI time course data, measured 24 h after transfection, via flow cytometric analysis of transfected cell populations during lentiviral production phase of the bioprocess. (E) Assessment of transfection efficiency, measured 24 h after transfection, and (F) functional HIV-1-GFP LVV titer when preparing liposomes at a pH of 6.65–6.80, prior to lipoplex formation. (G) Assessment of transfection efficiency, measured 24 h after transfection, and (H) functional HIV-1-GFP LVV titer when preparing liposomes at a pH of 6.40–6.60. Each data point (E−H) is the average of replicate conditions (n is indicated at the bottom of bars) and error bars are ±1 SD of the mean. Statistical pairwise comparisons have been presented as *p* values. Unmod. FS, Unmodified FreeStyle 293 Expression Medium.

Statistical models describing the effect of the formulation pH and liposome incubation time on transfection efficiency and MFI were constructed. Following analysis of the transfection efficiency (*p* < 0.0001) and median fluorescence intensity (MFI) (*p* = 0.0004) statistical models, a significant two-factor interaction between liposome formulation pH and the duration of nanoparticle incubation was determined. This indicated a combined effect of the two independent variables on the measured responses where the effect of one variable on the responses was not consistent across the different levels of the other variable. Model analysis revealed that peak particle performance occurred in a plane through the design space. The highest transfection efficiencies and MFIs were achieved when either using a high formulation pH (pH = 7.40) in combination with a short incubation time (5 min) or when utilizing a low formulation pH (pH = 6.80) in conjunction with a longer incubation time (30 min) (Figures 2A and 2B). The ability to achieve equivalent particle performance when operating in distinctly distant areas of the design space is likely attributable, in part, to the existence of an optimal particle size range of liposome particles prior to their complexion with pDNA. Earlier particle characterization experiments demonstrated that particle growth rates were dependent on the pH of the medium and particles were shown to grow at a decreased rate when incubated in formulations maintained at a lower pH set-point, meaning that they likely take longer to achieve the optimal size. Particles incubated at a pH of 7.40 for a 5-min period and at a pH of 6.80 for a 30-min period achieved sizes of approximately 850 nm and 1,150 nm, respectively (Figure 1A).

Since peak particle performance could be effectively delayed when preparing liposomes in low pH formulations, likely due to slowed particle growth kinetics, it was hypothesized that liposomal stability would be enhanced, ensuring that particles would be able to retain their ability to facilitate efficient transfection for prolonged incubation periods. To examine this, and to further characterize the relationship between formulation pH and liposome incubation time, a wider range of liposome incubation durations were investigated. Particles were incubated at both a pH of 7.40, which is within the specified pH range of FreeStyle 293 Expression Medium (7.0–7.6) and within the range widely considered to be optimal for mammalian cell culture medium, and at a pH of 6.75. Data from these experiments showed that the peak transfection efficiency achieved (51.6%) when preparing liposomes at a pH of 6.75 was attained after a 30-min incubation period (Figure 2C). This was higher than the maximum transfection efficiency achieved (42.9%) when liposomes were prepared at a pH of 7.40 and incubated for 5 min. A higher maximum MFI was also obtained when incubating liposomes at a pH of 6.75, determined to be 30% greater than the maximum MFI achieved when incubating liposomes at a pH of 7.40 (Figure 2D). Additionally, the incubation of liposomes at the lower pH setting of 6.75 was found to enhance the stability of the nanoparticles (Figures 2C and 2D), enabling the particles to retain their ability to facilitate efficient transfections for a prolonged period of time where similar transfection efficiencies were achieved following a 30- and 60-min incubation period. Conversely, the stability of liposomes prepared at the physiological pH of the culture medium was poor and peak particle performance, attained after a 5-min liposome incubation period, rapidly decreased when longer incubation periods were observed. Transfection efficiency decreased from 42.9% to 24.6% when the incubation time was increased from 5 min to 15 min, corresponding with a 67% decrease in the MFI (Figures 2C and 2D).

Based on the authors’ experience of preparing transfection mixtures for large-scale (>100 L) good manufacturing practice (GMP) manufacturing processes, it was determined that a liposome incubation period of 30–60 min would be a desirable duration for the intermediate liposome preparation phase of the lipoplex formulation procedure. It was important to define an appropriate pH operating range where the peak liposome particle performance was maintained for this extended incubation period. As such, a pH range of 6.40–6.80 was investigated. Excellent liposome stability was demonstrated when nanoparticles were incubated in formulations maintained at a pH of 6.40, 6.50, 6.60, 6.65, or 6.75 prior to liposome complexion with pDNA (Figures 2E–2H). Equivalent transfection efficiencies, MFIs and functional vector titers (*p* > 0.05) were obtained following 30- and 60-min incubation periods (Figures 2E–2H). However, significant decreases in transfection efficiency (*p* = 0.0061) and MFI (*p* = 0.0011) were detected between the 30- and 60-min incubation periods when liposomes were incubated at a pH of 6.80, demonstrating lower particle stability at the higher pH. This is likely attributable to the faster liposome growth rate under these conditions, where particles may have exceeded the optimal size range after a 60-min incubation period. Poor liposome stability was observed in the process control, where liposomes were incubated at a pH of 7.40, and a significant decrease in transfection efficiency and MFI were calculated between the 5- and 15-min incubation periods. Transfection efficiency decreased from 60% to 35% (*p* < 0.0001), corresponding with a 72% decrease in the relative level of transgene expression (*p* < 0.0001). Additionally, there was a 53% decrease in titer from 3.0 × 10^7^ TU/mL to 1.4 × 10^7^ TU/mL over this 10-min period (*p* = 0.0004).

**Figure 3:**
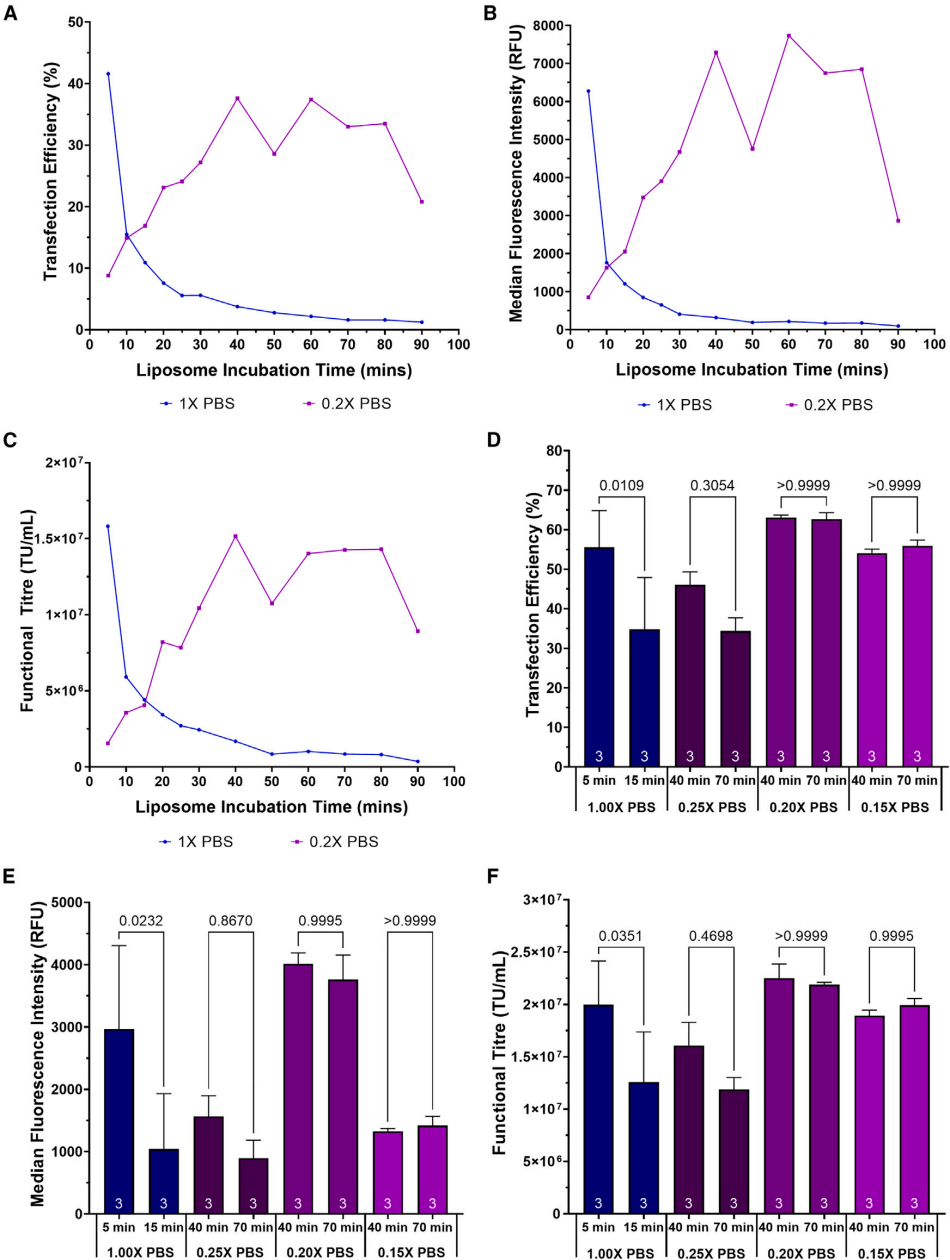
Assessment of liposome particle performance and stability when preparing nanoparticles in differing concentrations of PBS (A) Transfection efficiency and (B) MFI, measured 24 h after transfection, and (C) functional vector titer in the culture supernatants at harvest when preparing liposomes in differing concentration of PBS. (D) Assessment of transfection efficiency and (E) MFI, measured 24 h after transfection, and (F) functional HIV-1-GFP LVV titer when nanoparticles were prepared in diluted PBS formulations compared with 1X PBS. Each data point (D–F) represents the mean ±1 SD (n = 3). Statistical pairwise comparisons have been presented as *p* values.

To determine whether the salt concentration of formulations used to prepare liposomes was a key determinant of transfection efficiency, the ability of liposomes prepared in PBS formulations of varying ionic strengths to transfect cells in 24-deep well plates (DWPs) was investigated. The concentrations selected, 0.2× PBS and 1× PBS, were based on the differential liposome growth rates observed across this range (Figure 1E). Liposome incubation durations, ranging from 5 min to 90 min, were investigated. The maximum transfection efficiency achieved when preparing liposomes in a 1× PBS concentration (41.6%) was attained after a 5-min incubation period (Figure 3A). A comparable maximum transfection efficiency was obtained when liposomes were incubated in 0.2× PBS (37.6%) after a 40-min incubation period. This finding demonstrated that the onset of peak particle performance could be delayed when preparing liposomes in low salt concentration formulations. A higher maximum MFI was calculated when incubating liposomes at 0.2× PBS, determined to be 19% greater than the maximum MFI achieved when incubating liposomes at 1× PBS (Figure 3B). The incubation of liposomes in 0.2× PBS was additionally found to enhance liposome stability, enabling the particles to retain their ability to facilitate efficient transfections for prolonged periods of time compared with liposome preparation in higher salt concentrations (1× PBS). Comparable transfection efficiencies and MFIs were achieved following a 40-min to 80-min incubation for 0.2× PBS. Conversely, the stability of liposomes prepared in 1× PBS was poor and peak particle performance, attained after a 5-min liposome incubation period, rapidly decreased when longer incubation periods were observed. Transfection efficiency decreased from 41.6% to 10.9% when the incubation time was increased from 5 to 15 min, corresponding with a 72% and a 63% decrease in MFI and functional titer, respectively (Figures 3A–3C).

To verify the findings from the initial screening experiment, liposomes were prepared in 0.15×, 0.2×, and 0.25× PBS and incubated for 40 min or 70 min prior to their complexion with pDNA. The resulting lipoplexes were used to transfect suspension HEK293T cultures in E125 shake flasks. Improved liposome stability was demonstrated when nanoparticles were incubated in 0.15×, 0.20×, and 0.25× PBS formulations prior to liposome complexion with pDNA; equivalent transfection efficiencies, MFIs and functional vector titers (*p* > 0.05) were obtained following 40- and 70-min incubation periods, demonstrating a broad window of optimal operability (Figures 3D–3F). Conversely, poor liposome stability was observed when liposomes were incubated in 1× PBS and transfection efficiency declined from 55.6% to 34.8% (*p* = 0.0109) when the incubation time was increased from 5 min to 15 min. This corresponded with a 72% decrease in the MFI (*p* = 0.0232) and a 53% decrease in titer (*p* = 0.0351).

### Generation of high concentration lipoplex-based transfection mixtures, while maintaining transfection performance

Increasing the concentration of liposomes was initially shown to accelerate particle growth rates and increase particle size. This is undesirable, since these properties have been correlated with reduced particle performance. Liposome particle characterization studies revealed that liposome concentration, in addition to formulation pH and salt concentration, was a determinant of zeta-potential. We, therefore, hypothesized that liposomes could be prepared at significantly higher concentrations (in excess of an order of magnitude) than contemporary protocols, while ensuring particles could deliver equivalent levels of performance. This would be achieved by decreasing the volume of the diluent to a level that ensured a sufficiently large liposome zeta-potential to counteract the accelerated growth rates associated with nanoparticles being prepared at high concentrations.

**Figure 4:**
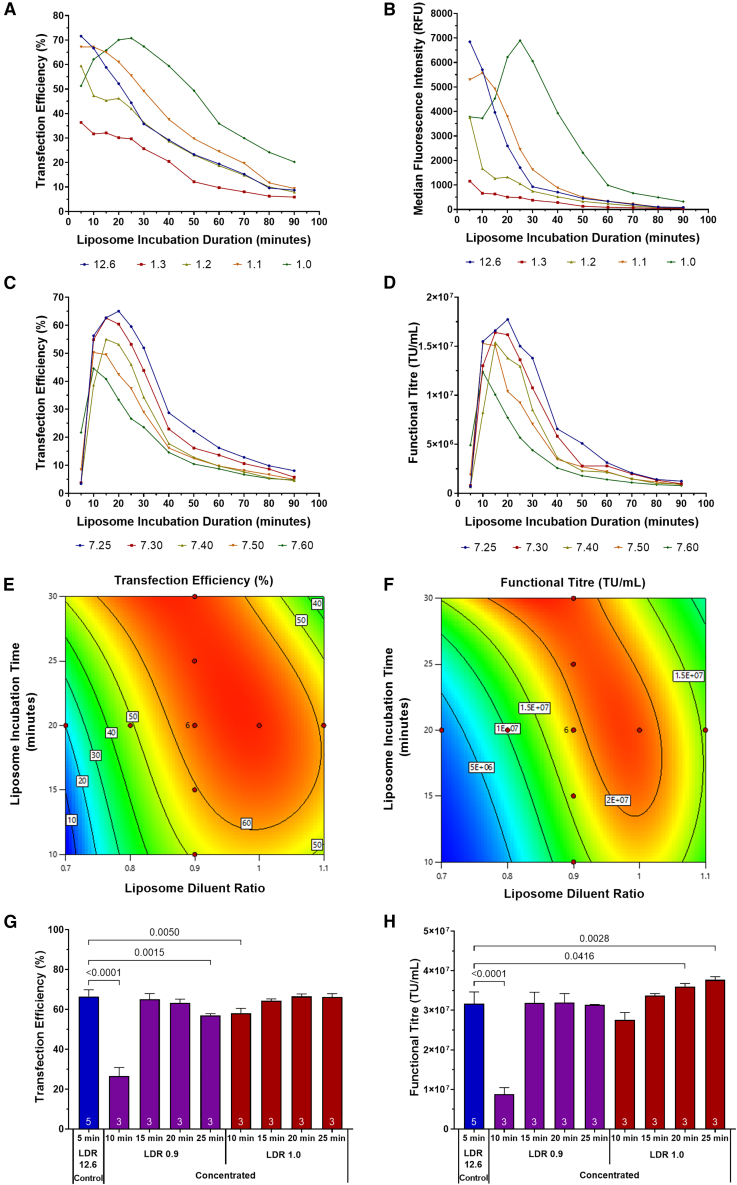
Development of high concentration lipoplex-based transfection formulations (A) Transfection efficiency and (B) MFI, measured 24 h after transfection of transfected cell populations during HIV-1-GFP LVV production phase of the bioprocess. Liposomes were prepared using variable diluent ratios to adjust the concentration of the resulting lipoplexes in the final transfection formulations. (C) Transfection efficiency, measured 24 h after transfection, and (D) functional LVV titers measured in conditions where liposomes were prepared using FreeStyle 293 Expression Medium adjusted and maintained at varying pH set-points prior to the formulation of lipoplexes. (E) Two-dimensional contour maps of the design space modeled for the impact of the liposome diluent ratio and liposome incubation time on transfection efficiency and (F) functional LVV titer. (G) Assessment of transfection efficiency, measured 24 h after transfection, and (H) functional HIV-1-GFP LVV titers when preparing lipoplexes at high concentrations by reducing liposome diluent ratios. LDR, liposome diluent ratio. Each data point is the average of replicate conditions (n is indicated at the bottom of bars) and error bars are ±1 SD of the mean. Statistical pairwise comparisons have been presented as *p* values.

To test this, liposomes were prepared in FreeStyle 293 Expression Medium, investigating a diluent to liposome ratio range of 12.6:1.0 to 1.0:1.0. A range of liposome incubation durations, ranging from 5 min to 90 min, were investigated to assess liposome particle performance over a prolonged incubation period. Results showed that the peak transfection efficiency and MFI achieved (71.6% and 6,847 relative fluorescence units [RFU], respectively) when preparing liposomes at a ratio of 12.6:1.0 after a 5-min incubation period, were similar to the maximum transfection efficiency and MFI achieved (70.8% and 6,899 RFU, respectively) when liposomes were prepared at a diluent ratio of 1.0:1.0 after a 25-min incubation period (Figures 4A and 4B). Reduced particle performance was observed in the conditions involving liposome preparation using higher volumes of diluent (ratios of 1.3:1.0, 1.2:1.0, and 1.1:1.0) likely attributed to reduced liposome zeta-potentials and accelerated particle growth rates under these conditions (Figures 1G–1I).

Since formulation pH has been identified as a key determinant of liposome zeta-potential and growth rates, the impact of this variable on the preparation of liposomes at high concentrations was assessed. Concentrated liposomes were incubated over a period of 90 min in FreeStyle maintained at pH values ranging from 7.25 to 7.60 (Figures 4C and 4D). In each preparation, the diluent to liposome ratio was fixed at a 0.9:1.0. A decrease in formulation pH from 7.60 to 7.25 correlated with an increase in the peak transfection efficiency achieved and also with a delay in the time point that peak efficiency was achieved in the respective incubation time courses. This is concordant with earlier particle characterization studies where lower pH values were found to correlate with larger zeta-potential values and, therefore, lower particle growth rates. The peak functional titers achieved in the conditions involving liposome incubation at pH 7.25 to 7.50 were similar despite higher transfection efficiencies being achieved at the lower pH values. A lower functional titer was obtained when using a pH of 7.60; however, it is possible that the peak may have occurred between the 5- to 10-min incubation period. A three-factor, five-level customized fcCCD response surface DoE was utilized to examine the impact of the diluent to liposome ratio, liposome incubation time, and formulation pH on transfection performance in HEK293T cultures cultivated in 24-DWP systems (Figures 4E and 4F). A significant two-factor interaction between the diluent to liposome ratio and liposome incubation time was determined following analysis of the transfection efficiency (*p* < 0.0001), MFI (*p* < 0.0001), and functional lentiviral vector (LVV) titer (*p* < 0.0001) models. Additionally, a significant two-factor interaction between diluent to liposome ratio and formulation pH was determined on transfection efficiency (*p* = 0.0080) and functional LVV titer (*p* = 0.0005). To maximise transfection efficiency and functional LVV titers, the DoE optimization identified that a diluent ratio of 0.95 should be used in combination with a liposome incubation time of approximately 20 min. When operating in this area of the design space, the models indicated that variations in pH between 7.25 and 7.60 had a minimal impact on particle performance.

To verify the findings from the DoE study, an additional experiment was conducted that involved the preparation of concentrated lipoplexes, compared with standard preparation methodologies, in replicate E125 shake flasks. Lipofectamine diluent ratios of 0.9:1.0 and 1.0:1.0 were employed in combination with liposome incubation periods of 10–25 min. Equivalent transfection efficiencies and functional LVV titers to the standard lipoplex formation strategy were obtained when using Lipofectamine diluent ratios of 0.9:1.0 and 1.0:1.0 in combination with an incubation period of 15 min and 20 min (Figures 4G and 4H).

### Scale-up of alternative lipoplex formulation methodologies

Following the identification of optimal lipoplex formulation methodologies that (1) delay the onset of peak particle performance, (2) enhance liposomal stability, and (3) enable highly concentrated lipoplex solutions to be generated, additional experiments were conducted in ambr 250HT bioreactors to verify that the benefits discerned at small scale were also observed in larger scale bioreactor systems. This was essential, since the alternative lipoplex formulation methods developed in this work seek to address manufacturing challenges that are specifically encountered when carrying out large-scale transient transfection unit operations.

**Figure 5:**
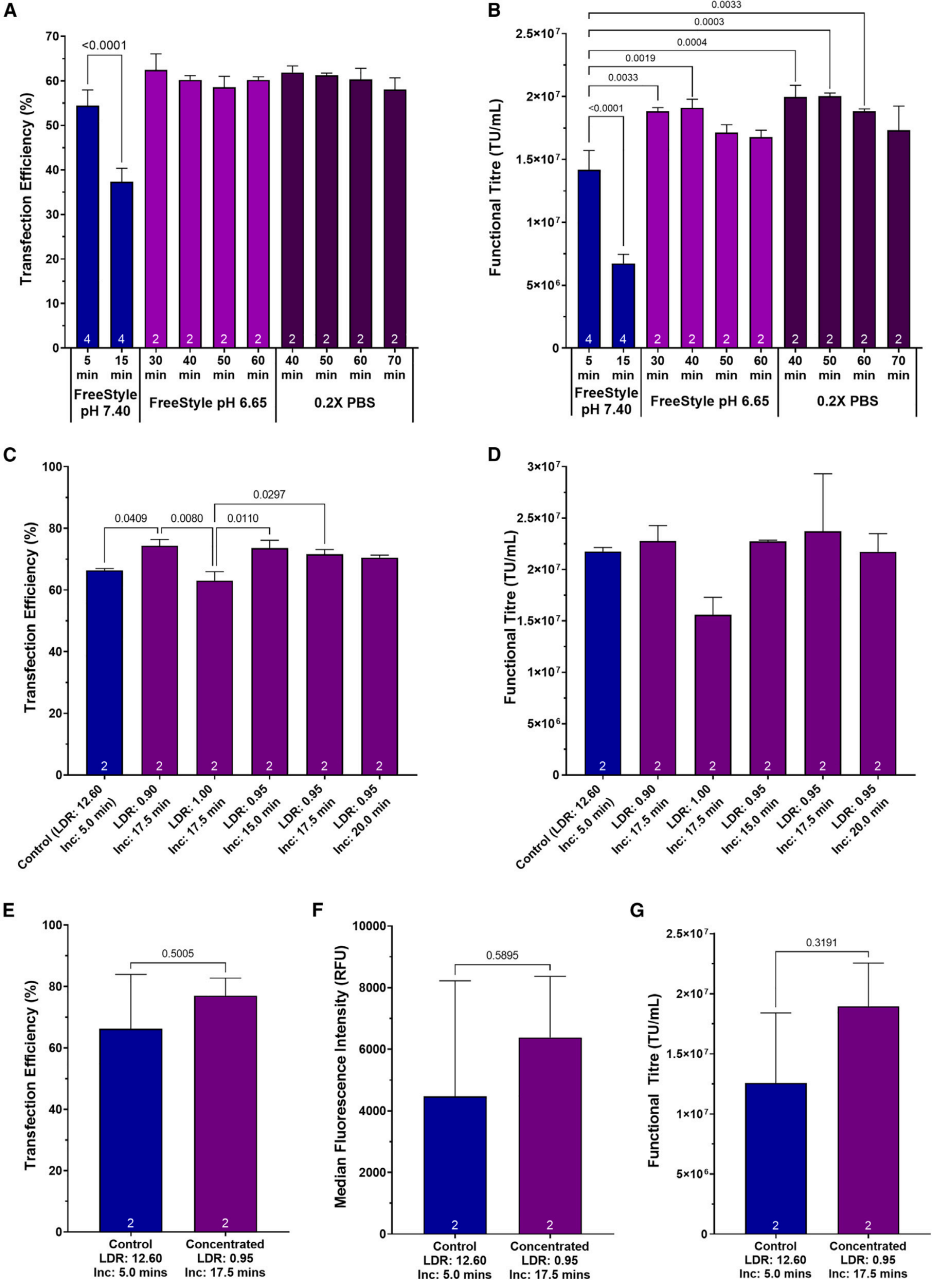
Assessment of the scalability of the alternative lipoplex formulation methodologies in ambr 250HT and 5-L scale bioreactors (A) Assessment of transfection efficiency, measured 24 h after transfection, and (B) functional HIV-1-GFP LVV titers when preparing lipoplexes in low pH and low salt concentration formulations in ambr 250HT bioreactors. (C) Assessment of transfection efficiency, measured 24 h after transfection, and (D) functional HIV-1-GFP LVV titers when preparing lipoplexes in high concentration formulations in ambr 250HT bioreactors. (E) Assessment of transfection efficiency and (F) median fluorescence intensity, measured 24 h after transfection, and (G) functional HIV-1-GFP LVV titer when preparing lipoplexes in a high concentration formulation in 5-L scale bioreactors. Each data point is the average of replicate conditions (n is indicated at the bottom of bars) and error bars are ±1 SD of the mean. Statistical pairwise comparisons have been presented as *p* values. Inc, incubation period; LDR, liposome diluent ratio.

Liposomes were prepared in either FreeStyle 293 Expression Medium maintained at a pH of 6.65 or in 0.2× PBS. Particles were incubated for periods of between 30–60 min and 40–70 min, respectively, prior to complexion with pDNA. Particle performance was compared with liposomes prepared via the manufacturer’s recommended transfection protocol. Once formulated, all liposomes were complexed with pDNA required to produce HIV-1-GFP LVVs and the resulting lipoplexes added to suspension HEK293T cultures cultivated in ambr 250HT bioreactors (operated at a working volume of 250 mL). Poor liposome stability was observed when preparing nanoparticles according to the standard preparation method, where increasing the liposome incubation time from the manufacturer’s recommendation of 5 min to a longer 15 min incubation was found to be detrimental to process performance, corresponding with a 31.4% decrease in transfection efficiency (*p* < 0.0001) and a 52.5% decrease in functional titer (*p* < 0.0001) (Figures 5A and 5B). This aligns with earlier observations in the smaller scale systems. Conversely, excellent stability was demonstrated when preparing liposomes according to the developed alternative methods and equivalent particle performance was obtained in all experimental conditions and across the full range of incubation periods with no significant differences being detected between the transfection efficiencies and functional titers achieved by the nanoparticles.

To verify that high concentration lipoplex-based transfection mixes could be prepared without compromising process performance, an additional experiment was conducted which involved the preparation of concentrated lipoplexes compared with standard preparation methodologies. FreeStyle 293: Lipofectamine 2000CD ratios of 0.90:1.00, 0.95:1.00, and 1.00:1.00 were used in combination with liposome incubation periods of 15 , 17.5, and 20 min. Equivalent functional LVV titers to the standard lipoplex formation strategy were obtained in all concentrated lipoplex conditions, demonstrating that up to a 14-fold decrease in the volume of the liposome diluent could be achieved compared with the existing process, while maintaining process performance (Figures 5C and 5D). To demonstrate that the preparation methods were suitable for larger scale applications, the concentrated liposome formulation method was selected for further scale-up into 5-L bioreactors. This method was selected for further scale-up, since it presented the additional advantage of reducing the overall volume of the transfection mix, as well as delaying the onset of peak particle performance, compared with the other two methods. A FreeStyle 293:Lipofectamine 2000CD ratio of 0.95:1.00 was used in combination with a liposome incubation period of 17.5 min, corresponding with a 13-fold decrease in the volume of the liposome diluent and a resulting 6-fold overall reduction in the volume of the final transfection mix, following complexion with pDNA. Equivalent transfection efficiencies, levels of GFP transgene expression and functional LVV titers were obtained compared with the standard lipoplex formation strategy, demonstrating successful scale-up of the method to 5-L culture volumes (Figures 5E–5G).

## Discussion

Considerable progress in the CGT field has been made possible by recent advances in manufacturing and TGE technologies, which have enabled the production of clinical and commercial grade viral vectors at large scale.^43^^,^^44^ Valued at US$355 million in 2022, the global viral vector manufacturing market is expected to reach US$1790 million by 2031.^45^ While this has resulted in large contract development and manufacturing organizations investing heavily in viral vector production, a number of considerable manufacturing challenges persist. While transient transfection remains the principal method of viral vector production, the ability to achieve reliable and efficient delivery of nucleic acid into cells is often cited at a major challenge due to the complications associated with scaling transient transfection-based processes.^24^^,^^46^^,^^47^ This study has discussed three major challenges of scaling such processes related to liposome incubation time, complex stability, and transfection mixture volume and has outlined alternative transfection protocols that can be adopted to circumvent such problems when preparing transfection mixtures for hundred liter scale or greater manufacturing processes, thereby opening up potentially larger scale production processes associated with a more favorable cost of goods.

Key parameters governing the transfectability of the lipoplexes, including size, structure, morphology, surface charge, and degree of DNA condensation, can be monitored and characterized via several analytical methodologies, including DLS, zeta-potential measurements, gel retardation assays, microscopy techniques, and nuclease resistance assays.^2^ The work presented here highlights the importance of characterizing the physical and electrochemical properties of liposomes and correlating these characteristics with particle performance. Zeta-potential has become a standard characterization technique to describe the surface charge of nanoparticles and potentially offers a valuable metric for in-process monitoring when preparing liposomes in a GMP manufacturing process. Liposomes were found to exhibit higher zeta-potentials when prepared in (1) formulations maintained at a lower pH, (2) formulations with a reduced ionic strength, and (3) when prepared at high concentrations. When prepared under these conditions, liposomes were characterized by increased zeta-potentials and slowed growth kinetics, compared with preparation following conventional methods. This corresponded with improved particle stability. Colloidal particle populations exhibiting zeta-potential values close to 0 mV typically exhibit low stability and are characterized by rapid coagulation due to the van der Waals attractive forces that act between adjacent particles.^38^ As the zeta-potential moves away from zero, there is a corresponding increase in particle stability due to increased repulsive electrostatic forces between liposomes, resulting in enhanced colloidal stability.^48^ This likely explains the faster growth rates that were observed when liposomes were prepared in formulations of increased ionic strength and increased pH, in the ranges investigated. This observation is in line with previous studies that have shown zeta-potential to be sensitive to changes in formulation pH; Smith et al.^49^ demonstrated a change in the zeta-potential of 1,2-dioleoyl-sn-glycero-3-phospho-L-serine (DOPS)-containing liposomes from −36 mV to −45 mV when the formulation pH was increased from 5.8 to 7.7.^50^ Similarly, a number of studies have reported the impact of ionic strength on the zeta-potential of liposomes^49^^,^^51^^,^^52^^,^^53^ with Smith et al.^49^ reporting the zeta-potential of negatively charged DOPS-containing liposomes increasing from −63 mV to −21 mV when increasing the concentration of sodium chloride from 0 mol/L to 0.05 mol/L, while maintaining solution a pH of at 7.6.^49^ Only a modest reduction in particle growth rate was observed when preparing liposomes at 1°C and 5°C, compared with 23°C, potentially due to slowed particle diffusion rates at the lower temperatures. Equivalent liposome zeta-potentials were measured between 1°C and 23°C, likely accounting for the lack of greater differences between the particle growth rates. Similarly, Wang et al.^54^ previously reported equivalent zeta-potentials between 1,2-dioleoyl-3-trimethylammonium-propane/1,2-dioleoyl-sn-glycerophosphoethanolamine-based liposomes across a range of temperatures. Transfection complex characterization studies are often carried out in simple buffer solutions, distinct from the formulations used for the preparation of complexes as part of *in vitro* transfection studies.^23^ This study highlights that differences in liposome zeta-potential and particle size can be attributed to relatively small changes in pH and ionic strength. It is, therefore, crucial that particle characterization experiments are only linked to experimental results when conducted in the same medium/buffer that is used for transfection experiments since the precise formulation influences the physiochemical properties and biological activity of complexes.

Many contemporary transfection preparation methodologies requiring an intermediate liposome preparation step involve the utilization of short liposome incubation periods prior to the complexion of liposomes with nucleic acids.^41^^,^^42^^,^^55^^,^^56^^,^^57^^,^^58^ In the case of Lipofectamine brand reagents, a liposome incubation period of 5 min is generally recommended under standard preparation conditions. While short liposome incubation periods are easily achievable and practical for the small-scale preparation of lipoplexes, short incubation periods are extremely challenging to achieve in the context of hundred liter scale LVV GMP manufacturing processes due to the considerable logistical challenges associated with the transfer of large liquid volumes required to prepare transfection mixtures.^59^^,^^60^^,^^61^ The attainment of sufficiently short liposome incubation periods is often not possible in larger scale manufacturing processes and longer incubation periods must be observed, at a detriment to process performance. It is apparent that, at a physiological pH, longer incubation times are associated with a significant decrease in particle performance, undesirable, since this results in suboptimal process performance. The significant improvement in the stability of liposomes when incubated in formulations maintained at a low pH (pH of 6.40–6.75) and in low ionic strength formulations (0.2× PBS), compared with standard preparation methodologies, are of considerable industrial value. These alternative preparation methodologies facilitate the setting of extended normal and proven acceptable process ranges of operability in large-scale manufacturing bioprocesses. This will enable improved process robustness and reduced batch-to-batch variability for a multifaceted and complex process step that is known to be highly sensitive. Additionally, the effective delay in the onset of peak particle performance means that longer incubation periods, which are easily achievable for manufacturing teams, can be used when preparing liposomes as part of industrial manufacturing scale bioprocesses. Cationic polymers are also widely used to mediate large-scale transfections, with PEI being recently used in a 2,000-L scale recombinant adeno-associated virus manufacturing process.^62^ Incubation times have also been shown to be crucial for governing polyplex growth kinetics following the complexion of PEI and nucleic acid; longer incubation times, in excess of 30 min, have been correlated with decreased transfection efficiency.^63^ Indeed, it has been recently shown that growth rates of PEI-based polyplexes can be significantly decreased by decreasing the ionic strength of the formulation used to prepare the nanoparticles providing greater control over the preparation of transfection mixtures, in a similar manner to our lipid-based transfection complexes,^64^ highlighting a potential broader applicability of the methods devised in this work to both lipid and non-lipid based transfection-based platforms.

Manufacturers also recommend that cationic lipids be prepared in proportionally high volumes of diluent when preparing lipoplexes, resulting in the generation of volumetrically large transfection mixtures. A large number of manufacturer’s protocols for cationic lipid-based transfection advise cationic lipid:diluent ratios of between 1:8 and 1:500, including those for Lipofectamine-based reagents, 293fectin, TransFectin, FuGene HD, LV-MAX, 293-Free, and TurboFect.^41^^,^^42^^,^^55^^,^^65^^,^^66^^,^^67^^,^^68^^,^^69^ Issues can be encountered when scaling up TGE processes where manual bulk mixing methods in large-scale processes, which are difficult to standardize, can result in poor particle size uniformity, particle aggregation, and variation between batches.^70^^,^^71^^,^^72^ Microfluidic devices can potentially address some of these issues and have recently gained a lot of interest to produce lipid nanoparticles due to their high reproducibility, precise control over particle sizes, and high throughput for formulation optimization.^73^ However, the ability of such methods to provide sufficient volumes of nanoparticles for industrial manufacturing-scale transfection processes is a current limitation.^74^ It is desirable to reduce the volume of transfection mixtures in large-scale manufacturing processes for three principle reasons: (1) smaller formulation volumes ensure a simpler and more reproducible preparation strategy for manufacturing teams, hypothesized to reduce batch-to-batch variability, (2) reduced transfection volumes facilitate the use of larger initial vessel inoculum volumes in suspension systems, which may be able to enhance the productivity of certain bioprocesses, and (3) smaller formulation volumes will decrease the number of single-use consumables required in GMP manufacturing processes, or associated equipment footprint, thereby helping to decrease operating costs. The data presented outline a method that can be used to prepare liposomes at higher concentrations than those achievable when following standard preparation protocols, while avoiding the undesirable accelerated particle growth rates that were associated with preparing particles in reduced volumes of diluent. This was achieved by decreasing the volume of the diluent to a level that ensured a sufficiently large liposome zeta-potential to counteract the accelerated growth rates associated with nanoparticles prepared at high concentrations. Lipofectamine 2000-based liposomes prepared under these conditions were able to be prepared in reduced diluent volumes, 10- to 25-fold smaller than the volume range recommended by the supplier, while maintaining equivalent transfection performance. Since transfection volumes frequently account for approximately 10% of the overall working volume of the cell culture in many bioprocesses, we anticipate a 6-fold decrease in the overall transfection volume from approximately 20 L to approximately 3.3 L for a 200-L scale manufacturing process. This is based on the 13-fold decrease in the liposome diluent volume and resulting 6-fold overall decrease in the final transfection volume (following complexion with pDNA) from approximately 0.50 L to approximately 0.08 L achieved in the 5-L scale experiment. Preparing liposomes at these higher concentrations had the added benefit of slowing particle growth kinetics such that particles achieved peak transfection performance after a 15–20 min incubation period, compared with a 5-min incubation period under standard conditions.

The alternative lipoplex formulation strategies devised in this work seek to address several existing issues that are encountered specifically when carrying out large-scale transient transfection unit operations. Therefore, it was necessary to demonstrate that these solutions could be successfully scaled-up from 24-DWPs and E125 shake flasks, used in screening and optimization studies, into larger bioreactor systems. The three alternative liposome formulation methods described in this report were all successfully scaled into ambr 250HT bioreactors and the concentrated lipoplex formulation method, selected for further scale-up, was successfully scaled into 5-L bioreactors. The benefits of improved complex stability and the ability to effectively concentrate transfection mixtures, observed in smaller scale screening and optimization studies, were demonstrated in larger bioreactor systems. The ambr 250HT is used widely throughout the bioprocessing industry as an effective scale-down model for a range of different biological applications and products.^75^^,^^76^^,^^77^^,^^78^ Therefore, it is anticipated that the successful deployment of the described alternative lipoplex formulation strategies at ambr 250HT scale likely indicates that these methods would be successfully deployed at 50 L and 200 L scale. However, suitable experiments would need to be conducted to verify this.

Lipofectamine 2000CD was used a model transfection reagent in this work to demonstrate that alternative transfection protocols, more suitable for the large-scale generation of liposome-based transfection complexes, can be devised. It is anticipated that the methods outlined will be applicable to other transfection systems, both lipid based and cationic polymer based, but further optimization work will likely be required to account for the specific chemistries of other reagents. It should also be acknowledged that the methods derived in this study may have applications outside of viral vector production and may be useful for lipoplex preparation methods that can be employed in other therapeutic applications, such as cancer treatments.^79^ The liposome preparation methodologies developed as part of this work are intended to ensure process scalability and conformity with large-scale GMP manufacturing processes. The strategies outlined facilitated superior control over nanoparticle growth kinetics and resulted in enhanced particle stability; particles retained the ability to facilitate efficient transfections for prolonged periods of time compared with contemporary preparation methodologies. This has significant industrial applications for the large, hundred liter and above, scale manufacture of biologics, including LVVs, via transient transfection for two principal reasons. First, it enables longer incubation times, practically achievable when manufacturing LVVs at large scale, to be used since the onset of peak particle performance can be effectively delayed. Second, the significant improvement in particle stability facilitates the setting of wider process operating ranges for a critical and highly sensitive process step. It is anticipated that this will significantly improve process robustness and minimize batch-to-batch variability for maximal process control.

## Materials and methods

### Cell culture

Suspension-adapted HEK293T cells, provided by Oxford Biomedica Limited, were routinely passaged (sub-cultured) in, serum-free, FreeStyle 293 Expression Medium (Gibco, Thermo Fisher Scientific) and maintained in a shaking incubator (orbital shaking diameter of 25 mm) at 37°C, 300 rpm and 5% CO_2_. Cells were cultivated in 24-DWPs, Erlenmeyer shake flasks, ambr 250HT (Sartorius AG) bioreactors, and 5-L scale stirred tank bioreactors (Applikon Biotechnology), at working volumes of 3 mL, 25 mL, 250 mL, and 5,000 mL respectively. ambr 250HT bioreactors (mammalian vessel configuration) and 5-L bioreactors were operated at 37°C, between a pH of 6.90 and 7.40 and at an agitation rate of 600 rpm and 300 rpm, respectively. Cell culture medium osmolality was measured using an Osmotech Single Sample Micro Osmometer (Advanced Instruments).

### LVV production

Recombinant, pseudotyped, replication-incompetent LVVs were produced using Oxford Biomedica’s propriety LentiVector delivery platform components. Briefly, HIV-1-based LVVs were produced via the transient co-transfection of suspension-adapted HEK293T cells with third-generation packaging plasmids encoding HIV helper function, an envelope plasmid construct encoding the vesicular stomatitis virus-G protein and a vector genome transfer plasmid encoding GFP. Four plasmids were co-transfected in total. Cells were transfected with lipoplexes, prepared via the combination of the required plasmids complexed with the cationic lipid, Lipofectamine 2000CD (Invitrogen, Thermo Fisher Scientific). Transfection mixtures were prepared under ambient conditions and cultures were transfected at cell densities of 2 × 10^6^ viable cells/mL. Transfection volumes were approximately 10% of the total working volume at each scale and were manually prepared and added to cultures at all scales. When preparing transfection mixtures via the concentrated liposome formulation method, the pDNA fraction and the liposome fraction were concentrated so that both existed in equal volumes, ensuring good mixing. Mixing and addition of transfection complexes was done according to the manufacturer’s guidelines. Transfected cell populations were supplemented with sodium butyrate (Sigma-Aldrich, Merck), and the HIV-1-based LVV containing supernatant was isolated, clarified through a 0.45-μm filter and stored at −80°C for subsequent analysis.

### DLS and zeta-potential analysis

DLS and zeta-potential analysis of samples was performed with a Zetasizer Nano particle analyzer (Malvern Scientific) at 23°C. Particle size was determined by measuring the Brownian motion of particles in samples, using DLS, and interpreting particle size using established theories. Size distributions of particles were generated via measuring back-scattered light (173° detection optics) of particles interrogated with a 633-nm laser. Each measurement was carried out in triplicate. The mean hydrodynamic diameters were evaluated as a *Z*-average using an automated data analysis mode. Zeta-potential measurements were performed using electrophoretic mobility measurements using laser Doppler velocimetry. Each zeta-potential result represents the mean of three readings. Dispersion Technology Software (Malvern Scientific) was used to collect all data. The particle size and polydispersity index were considered when interpreting data. All samples were prepared at room temperature.

### Transfection efficiency analysis

HEK293T cells were removed from vector production cultures approximately 24 h after transfection and populations were analyzed with a 488-nm excitation laser using an Attune NxT acoustic focusing flow cytometer (Thermo Fisher Scientific). Analysis was terminated when 10,000 live cell events had been processed. Subsequent data analysis was performed using FlowJo (FlowJo LLC) and transfection efficiencies were determined using the following equation:

(Equation 1) $$Transfection\;efficiency\;\left(\%\right)=\frac{Gated\;single,live,transgene\;positive\;cells}{Gated\;single,live\;cells}\times 100$$

The reported MFI of samples is a measure of the intensity of GFP expression for the entire live cell population and was calculated by multiplying the MFI of the gaited transgene positive cell population by the percentage of transgene-positive cells:

(Equation 2) $$MFI\;for\;total\;cell\;population=MFI\;of\;gated\;single,live,trangene\;positive\;cells\times \frac{Transfection\;Efficiency\;\left(\%\right)}{100}$$

MFI was used as a proxy of the relative levels of transgene expression between different cell populations.

### Functional vector titer

Functional vector titers were determined via the transduction of adherent HEK293T cells with HIV-1-based LVV particles, following the serial, 400-fold, dilution of vector preparations in DMEM (Sigma-Aldrich, Merck) supplemented with 8 μg/mL polybrene (Sigma-Aldrich, Merck). Two replicate wells of adherent cells were transduced with each sample analyzed. Transduced cells were harvested 72 h following their exposure to the diluted viral vector preparations and subjected to analysis via flow cytometry using an Attune NxT acoustic focusing flow cytometer. The total number of single, live, transgene positive cells as a percentage of the total number of single, live cells was determined via analysis of the cell populations on FlowJo. Assays were deemed valid if transduced cell populations exhibited a total percentage of GFP-positive cells of less than 30% for each sample. Equation 3 was used to determine the concentration of functional transducing units/mL:

(Equation 3) $$Titer\;\left(TU/mL\right)=\frac{\left[\frac{\%\;of\;transgene\;positive\;cells\;}{100}\times Number\;of\;cells\;prior\;to\;transduction\times Dilution\;factor\right]}{Volume\;of\;vector\;added\;\left(mL\right)}$$

### Model construction and statistical analysis

Statistical models were built using Design Expert 13 (Stat-Ease). Datasets were transformed if required and predictive models for experimental responses were built using ANOVA. Insignificant model terms, and terms not required to support model hierarchy, were removed from models via an algorithmic, stepwise, backward elimination methodology based on a *p* value criterion of 0.05. Diagnostic tests were utilized to determine which mathematical model was applied to each measured response. Differences between means were evaluated using one-way ANOVA and post hoc multiple comparison tests (Tukey tests). Statistical tests were performed using GraphPad Prism V9.1 (GraphPad Software) and values considered to be statistically significant when *p* values were less than 0.05 (∗), 0.01 (∗∗), 0.001 (∗∗∗), and 0.0001 (∗∗∗∗).

## Data and code availability

The data that support the findings of this study are included in the article or are available from the corresponding author upon reasonable request.

## Supplemental information

Document S1. Figure S1

Document S2. Article plus supplemental information
