# Supplementary material for: Development of novel lipoplex formulation methodologies to improve large-scale transient transfection for lentiviral vector manufacture
Source: Mol Ther Methods Clin Dev. 2024 Apr 26;32(2):101260. doi: 10.1016/j.omtm.2024.101260 (PMC11092396; doi:10.1016/j.omtm.2024.101260)
Supplement: Document S1. Figure S1 [file mmc1.pdf]

OMTM, Volume *32*

Supplemental information

Development of novel lipoplex formulation methodologies to improve large-scale transient transfection for lentiviral vector manufacture

Thomas Williams-Fegredo, Lee Davies, Carol Knevelman, Kyriacos Mitrophanous, James Miskin, and Qasim A. Rafiq

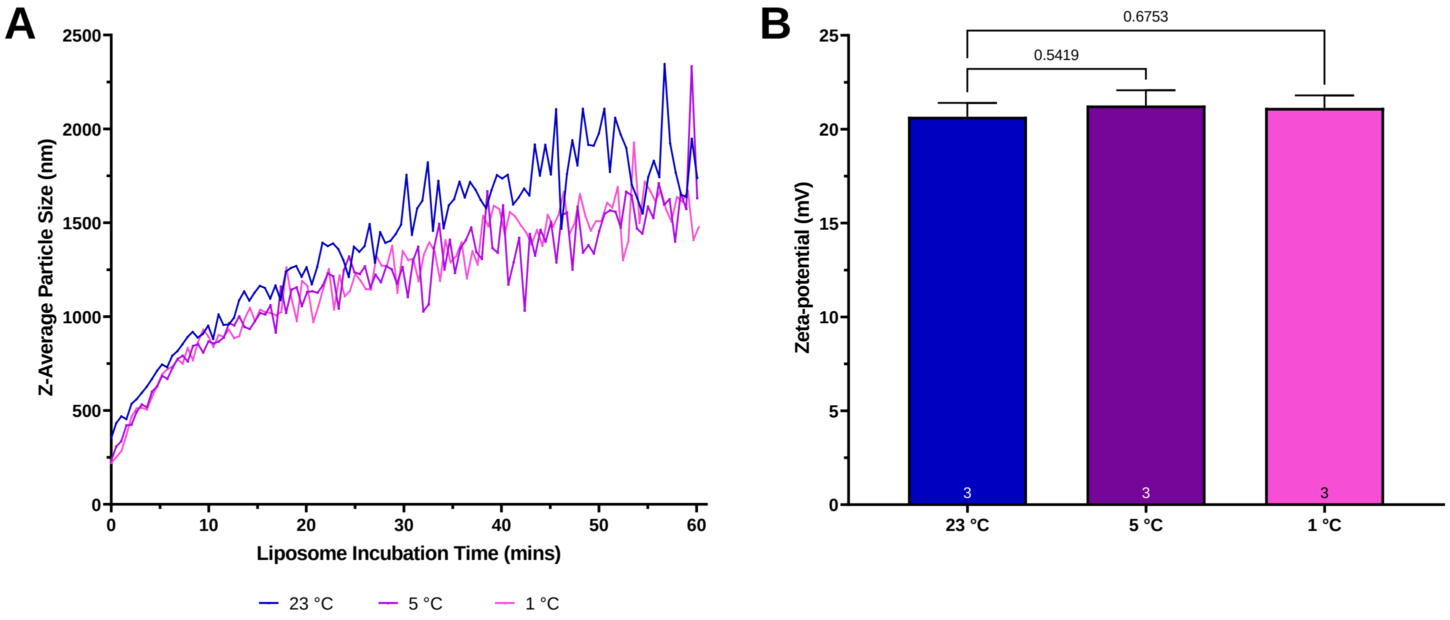

Figure S1: Lipofectamine™ 2000CD liposome particle characterisation at different temperatures, related to Figure 1. **(A)** Liposome growth profiles measured in FreeStyle 293 Expression Medium maintained between 1 °C to 23 °C. **(B)** Liposome zeta-potential measured between 1 °C to 23 °C. Data represents the mean ± one standard deviation (n = 3, replicates indicated at bottom of bars) and statistical pairwise comparisons have been presented as p values.
